# Visceral adiposity in postmenopausal women is associated with a pro-inflammatory gut microbiome and immunogenic metabolic endotoxemia

**DOI:** 10.1186/s40168-024-01901-1

**Published:** 2024-10-04

**Authors:** Mohamed Gaber, Adam S. Wilson, Amy E. Millen, Kathleen M. Hovey, Michael J. LaMonte, Jean Wactawski-Wende, Heather M. Ochs-Balcom, Katherine L. Cook

**Affiliations:** 1 Department of Surgery, Wake Forest University School of Medicine, Winston-Salem, NC 27157 USA; 2 Department of Cancer Biology, Wake Forest University School of Medicine, Winston-Salem, NC 27157 USA; 3 Department of Epidemiology and Environmental Health, School of Public Health and Health Professions, University at Buffalo, The State University of New York, Buffalo, NY 14214 USA

**Keywords:** Obesity, Menopause, Aging, Women’s Health Initiative, Microbiome, Inflammation, Lipopolysaccharide, Leaky gut, Metabolic endotoxemia

## Abstract

**Background:**

Obesity, and in particular abdominal obesity, is associated with an increased risk of developing a variety of chronic diseases. Obesity, aging, and menopause are each associated with differential shifts in the gut microbiome. Obesity causes chronic low-grade inflammation due to increased lipopolysaccharide (LPS) levels which is termed “metabolic endotoxemia.” We examined the association of visceral adiposity tissue (VAT) area, circulating endotoxemia markers, and the gut bacterial microbiome in a cohort of aged postmenopausal women.

**Methods:**

Fifty postmenopausal women (mean age 78.8 ± 5.3 years) who had existing adipose measurements via dual x-ray absorptiometry (DXA) were selected from the extremes of VAT: *n* = 25 with low VAT area (45.6 ± 12.5 cm^2^) and *n* = 25 with high VAT area (177.5 ± 31.3 cm^2^). Dietary intake used to estimate the Healthy Eating Index (HEI) score was assessed with a food frequency questionnaire. Plasma LPS, LPS-binding protein (LBP), anti-LPS antibodies, anti-flagellin antibodies, and anti-lipoteichoic acid (LTA) antibodies were measured by ELISA. Metagenomic sequencing was performed on fecal DNA. Female C57BL/6 mice consuming a high-fat or low-fat diet were treated with 0.4 mg/kg diet-derived fecal isolated LPS modeling metabolic endotoxemia, and metabolic outcomes were measured after 6 weeks.

**Results:**

Women in the high VAT group showed increased Proteobacteria abundance and a lower Firmicutes/Bacteroidetes ratio. Plasma LBP concentration was positively associated with VAT area. Plasma anti-LPS, anti-LTA, and anti-flagellin IgA antibodies were significantly correlated with adiposity measurements. Women with high VAT showed significantly elevated LPS-expressing bacteria compared to low VAT women. Gut bacterial species that showed significant associations with both adiposity and inflammation (anti-LPS IgA and LBP) were Proteobacteria (*Escherichia coli*, *Shigella* spp., and *Klebsiella* spp.) and *Veillonella atypica*. Healthy eating index (HEI) scores negatively correlated with % body fat and anti-LPS IgA antibodies levels. Preclinical murine model showed that high-fat diet-fed mice administered a low-fat diet fecal-derived LPS displayed reduced body weight, decreased % body fat, and improved glucose tolerance test parameters when compared with saline-injected or high-fat diet fecal-derived LPS-treated groups consuming a high-fat diet.

**Conclusions:**

Increased VAT in postmenopausal women is associated with elevated gut Proteobacteria abundance and immunogenic metabolic endotoxemia markers. Low-fat diet-derived fecal-isolated LPS improved metabolic parameters in high-fat diet-fed mice giving mechanistic insights into potential pro-health signaling mediated by under-acylated LPS isoforms.

Video Abstract

**Supplementary Information:**

The online version contains supplementary material available at 10.1186/s40168-024-01901-1.

## Introduction

In the US and other developed countries, obesity is recognized as a major public health problem. In 2017–2018, the prevalence of obesity in US adults (≥ 20 years of age) was 42.4% [1], representing a continued trend in increasing prevalence over the past two decades. Obesity, and in particular abdominal obesity, is associated with several comorbid conditions that share underlying inflammatory mechanisms. Over the past decade, studies have identified microbial correlates of obesity in humans [2] and animal models [3, 4] to help improve the understanding of host-microbiome relationships and metabolic consequences.

The gut microbiome is recognized to play an important role in the development of the host immune system and vice versa [5]. Several immune functions have been attributed to the gut microbiome including the activation and differentiation of T cells and B cells, activation of macrophages, and the modulation of neutrophil migration and function [6]. Reciprocally, the immune system is responsible for maintaining a delicate balance between elimination of pathogenic microbes and preserving beneficial commensals [7]. The gut microbiome also plays a critical role in metabolic health by increasing insulin sensitivity, improving glucose tolerance, and increasing energy expenditure [8, 9]. Hence, accumulating evidence demonstrates the implication of gut dysbiosis in metabolic diseases such as obesity and type 2 diabetes [8]. Gut microbiome dysbiosis is related to alterations in the gut permeability barrier and a local pro-inflammatory response [5]. As the body of knowledge connecting the microbiome to immunity and metabolic health later in life continues to develop [10], it is critical to evaluate potentially differential mechanisms through which microbiome shifts occur and whether they are age and/or obesity related. Aging is known to cause chronic low-grade systemic inflammation in a phenomenon known as “inflammaging” which might synergize with local gut inflammation in promoting gut dysbiosis and a pro-obesity milieu [11]. A few studies have investigated the impact of aging on the gut microbiome, especially with regard to women’s health and menopause. As women go through the menopausal transition, they are more likely to gain weight, and their gut microbiome composition shifts, implicating a pivotal role of sex hormones in shaping the gut microbiome [12]. This is further supported by the absence of sex differences in the gut microbiome before puberty [13]. In addition to sex hormones, the gut microbiome is modulated independently by aging [14] and obesity [15]. However, if, and how, obesity shifts the gut microbiome in elderly postmenopausal women is under explored and may represent specific interactions between sex, aging, and obesity.

It is hypothesized that the gut microbiome may contribute to the development of abdominal obesity [16], adding to the list of known obesity-associated factors including inherited genetic susceptibility, diet, and physical inactivity. Here, we evaluated the association of the gut microbiome, biomarkers of gut microbiome-induced systemic inflammation, and abdominal adiposity in a sample of elderly postmenopausal women using a cross-sectional design to study inflammatory links. We compare fecal lipopolysaccharides (LPS)-expressing microbe abundance in women with high visceral adipose tissue (VAT) area and low VAT area groups, as well as plasma LPS concentrations, LPS-binding protein (LBP) concentrations, anti-LPS IgA abundance, and anti-LPS IgG abundance. Furthermore, we expanded the study to determine adiposity associations with plasma anti lipoteichoic acid (LTA) IgA, anti-LTA IgG, anti-flagellin IgA, and anti-flagellin IgG abundance. Finally, to demonstrate mechanistic insights, we show differences in the metabolic outcomes of diet-derived fecal LPS between high-fat diet-fed mice and low-fat diet-fed mice. We show high-fat diet-fed female mice administered a low-fat diet-derived LPS reduced body fat composition and improved glucose homeostasis when compared with saline (control) and high-fat diet-derived LPS-treated mice. Ultimately, this work can advance our understanding of healthy aging in postmenopausal women and reduce the negative metabolic consequences of obesity-related gut dysbiosis.

## Material and methods

### Participant selection

The Buffalo Osteoporosis and Periodontal Disease (OsteoPerio) study is an ancillary study to the Women’s Health Initiative Observational Study (WHI-OS) [17]. Approximately, 2249 postmenopausal women were enrolled in the observational study of the WHI at the University at Buffalo from 1993 to 1998. Women were then further recruited into the Buffalo OsteoPerio study between 1997 and 2000. Women were excluded if they had fewer than six teeth, a bilateral hip replacement, a history of bone disease other than osteoporosis, a history of cancer within the previous 10 years, or treatment for serious illness. In total, 1362 women enrolled and completed the baseline visit [18]. These women were followed up over time including a visit 17-year post baseline where the focus of the study included the microbiome.

**Fig. 1:**
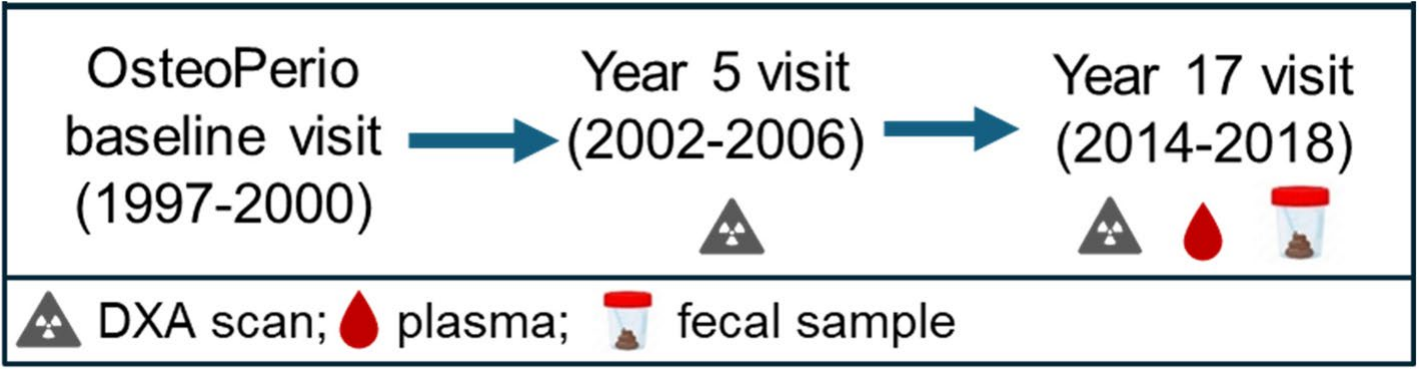
OsteoPerio study visits and sample collection

For the current study, participants were women in the OsteoPerio study that had available fecal and plasma samples at year-17 visit and dual x-ray absorptiometry (DXA) scans at both the year-5 (2002–2006) and year-17 (2014–2018) OsteoPerio study visit (*n* = 358, see Fig. 1, [18]). Of these participants, we excluded women with a history of hypertension, history of diabetes, reported hormone therapy usage (in past 90 days), and/or reported antibiotic usage (in the past 90 days) to remove potential medication interactions on the gut microbiome (*n* = 114). From these, we selected 50 participants (*n* = 25 low VAT area (45.6 ± 12.5 cm^2^) and *n* = 25 high VAT area (177.5 ± 31.3 cm^2^)) based on VAT quartiles in these women. See Supplemental Figure S1 for VAT area distribution in *n* = 114 eligible participants. Untargeted metabolomics (Metabolon; Raleigh, NC, USA) was performed on plasma samples collected at the same study visit (year-17) to evaluate/confirm antibiotic use. Three participants had detectable levels of antibiotics in plasma and were excluded from the analysis (Supplemental Figure S2).

The study was reviewed and approved by the Health Sciences Institutional Review Board at the University at Buffalo (Buffalo, NY; IRB no.: 030–581160), and all participants provided informed written consent.

### Anthropometric and adiposity measurements

Height was measured to the nearest 0.1 cm using a wall-mounted stadiometer. Weight was measured to the nearest 0.1 kg on a calibrated balance beam scale. BMI was calculated as weight (kg)/height^2^ (m^2^). All participants had a whole-body DXA scan at the year-17 study visit (Discovery A; Hologic Inc., Bedford, MA, USA) according to a standardized protocol by a trained and certified DXA technician.

Whole-body DXA scans were used to estimate total body fat mass (TBF, kg) and percent total body fat (TBF, %) [19]. To estimate abdominal VAT (cm^2^) and subcutaneous adipose tissue (SAT; cm^2^), DXA images were reanalyzed using new software (Hologic APEX 4.0 software toolbox). The proprietary procedures outlined in the Hologic Operator Manual (MAN-03644 Revision 005) were used to estimate VAT and SAT area in an abdominal region of interest 5 cm wide at approximately the 4th lumbar vertebrae, avoiding the iliac crest and limiting bony interference with the soft tissue measures.

Standardized procedures for participant positioning and scan analysis were used. Daily phantom scans and a random sampling of scans were reviewed to monitor machine and technician performance.

### Demographics, medication use, and diet

Self-administered questionnaires at year-17 visit were used to collect demographics (age, self-identified race, ethnicity, education, marital status), smoking habits, hormone therapy (HT) use, medications, and medical history [18]. Medications taken by patients were ascertained by inventory, and details were recorded directly from bottles. A validated, semiquantitative food-frequency questionnaire (FFQ) designed specifically for postmenopausal women [20, 21] was completed by all participants. Participants recalled dietary intake over the past 3 months, including > 350 unique foods.

The Healthy Eating Index 2015 (HEI-2015), a diet quality score ranging from 0 to 100 (higher scores indicating higher-quality diets), was scored for each participant using the nutrient and MyPyramid equivalents data [22, 23].

### DNA isolation and metagenomic sequencing

Fecal sample collections were performed at home using a stool collection kit. Participants were mailed a collection kit with instructions. They were instructed to place the sample into a collection tube with 4 ml of RNAlater, were instructed to refrigerate the sample, and bring the sample on ice to the clinic visit within 24 h of collection. Samples were then mixed and aliquoted to cryovials and stored at − 80 °C [17]. DNA was isolated from frozen fecal samples using the Qiagen DNeasy PowerLyzer PowerSoil Kit (Valencia, CA, USA), and 12-M read depth shotgun metagenomic sequencing was performed by CosmosID Inc. (Rockville, MD, USA). As previously performed [24], DNA libraries were prepared using the Illumina Nextera XT Library Preparation kit (San Diego, CA, USA), with a modified protocol [25, 26]. Library quantity was assessed with a Qubit fluorometer (Thermo Fisher Scientific, Wilmington, DE, USA). Libraries were then sequenced on an Illumina HiSeq platform to generate 150-bp paired-end reads. Unassembled sequencing reads (≥ 12-M read depth; see Supplemental Figure S3 for individual sample read statistics) were analyzed using the CosmosID bioinformatics platform described elsewhere [27–30] for multi-kingdom microbiome analysis, profiling of virulence genes, and quantification of microbial relative abundance.

### Determination of LPS-containing *bacteria*

Microbiota identified by shotgun metagenomic sequencing were annotated for LPS expression. Publicly available data on gene expression for each bacterium was used to determine LPS expression (in particular, the immunostimulant lipid A portion). The expression of *lpxA* and *lpxB* genes which encode the enzymes acyl-UDP-N-acetylglucosamine O-acyltransferase and lipid-A-disaccharide synthase, respectively, were used as determinants of LPS expression. Gene expression data was available for 83.3% of the identified bacteria on UniProt database (https://www.uniprot.org/), while the rest was determined via literature search.

### Plasma inflammation marker assessment

Fasting blood samples were obtained during the study visit by a trained phlebotomist. Samples were then processed within 90 min and stored at − 80 °C in cryovials. Plasma samples were used to measure gut inflammation and permeability markers. LPS levels were measured in diluted plasma samples (1:3) using the commercially available LPS Sandwich ELISA kit (LSBio, cat. no. LS-F17912) according to the manufacturer’s instructions. LBP levels were measured in diluted plasma samples (1:600) using the commercially available Human LBP ELISA kit (Invitrogen, cat. no. EH297RB). The average of duplicate readings was calculated for each sample, standard, and blank.

Measurement of IgA and IgG antibodies against LPS, LTA, and flagellin was done using lab-assembled kit as described previously [31–33]. Briefly, each plate well was coated with 50 µl of 40 µg/ml LPS (Sigma, cat. no. L2630) or 40 µg/ml LTA (InvivoGen, cat. no. tlrl-pslta) or 1 µg/ml flagellin (InvivoGen, cat. no. tlrl-stfla) in 50-mM carbonate/bicarbonate buffer (pH 9.6) overnight at 4 °C. After blocking step, diluted plasma samples in PBS (1:1200) were added to each well and incubated for 2 h at room temperature. After washing, 100 µl of anti-IgG (KPL, cat. no. 5220–0330) or anti-IgA (KPL, cat. no. 5220–0360) solutions diluted in PBS at 1:5000 and 1:2000, respectively, was added to each well. After washing, 90 µl of TMB substrate solution was added for 5–10 min (Sigma, cat. no. S5814). A total of 50 µl of stop solution was immediately added, and absorbance was measured at 450 nm. Absorbance at 540 nm was subtracted to correct for optical imperfections in the plates. The average of triplicate readings was calculated for each sample.

### C57BL/6 metabolic endotoxemia model

Female 7-week-old C57BL/6 mice (*n* = 48) were purchased from Jackson Laboratory and placed on a low-fat (10% kcal from fat, standard rodent chow) or high-fat diet (60% kcal from fat; Envigo, TD.06414) for 3 weeks. Approximately, 5 g of fecal matter was collected and pooled from 5 female C57BL/6 mice consuming the low-fat or high-fat diet for at least 6 weeks to generate diet-derived fecal LPS. LPS was isolated from fresh fecal material using LPS isolation kits (Sigma-Aldrich; MAK-339), and LPS concentrations were calculated (Sigma-Aldrich; MAK-104). Mice on each diet were randomized into 2 × weekly IP injections of saline (control), 0.4 mg/kg low-fat diet-derived fecal isolated LPS (LF-LPS), or 0.4 mg/kg high-fat diet-derived fecal isolated LPS (HF-LPS) for 6 weeks (*n* = 5–8 per group). LPS dose of 0.4 mg/kg experimentally represents metabolic endotoxemia [34]. Body weight was measured weekly. Echo-MRI was performed at the end of the study to determine % body fat composition. Glucose tolerance testing was performed at the end of the study as previously described [35]. This animal study was approved by the Wake Forest University School of Medicine Institutional Animal Care and Use Committee, and all procedures were carried out in accordance with relevant guidelines and regulations.

### Statistical analysis

Principal coordinates analysis (PCoA) of bacterial beta diversity based on the Bray–Curtis dissimilarity matrix using relative abundance was used to distinguish groups. Significance was assessed by PERMANOVA. Diversity analyses were performed using species taxonomy level. A Student’s *t*-test was used to measure significance in *α*-diversity. A Mann–Whitney test with *p* < 0.05 was used to determine significance in phyla, genus, and species proportional abundance. An unpaired *t*-test with Welch’s correction was used to compare plasma inflammatory markers by VAT groups. Correlations between anthropometric, plasma gut inflammatory markers, and bacterial species were summarized using Pearson’s correlation coefficients via GraphPad Prism 9 software.

## Results

Postmenopausal women in the sample had a mean age of 79.3 ± 6.3 years and 78.5 ± 4.2 years in the low and high VAT groups, respectively, and this difference was not statistically significant (Table 1). Women in the high VAT group had an approximate 7.5 kg/m^2^ higher BMI (*p* < 0.001), a 22.4 cm larger waist circumference (*p* < 0.001), and a 131.9 cm^2^ larger VAT area (*p* < 0.001) compared to the low VAT group. There was no difference in total energy intake (kcal/day) between the groups. There was a trending increase in mean HEI score in low VAT group compared to high VAT group (*p* = 0.051). Metabolomic analysis identified three participants with detectable antibiotics in plasma that were excluded from subsequent analysis (Supplemental Figure S2), resulting in a sample size of *n* = 47 for the remaining analysis.

**Table 1:** Participants’ characteristics at year 17 OsteoPerio study visit

Mean ± SD [Range]	**Low VAT group (*N* = 25)**	**High VAT group (*N* = 25)**	*p*-value*
**Age, y**	79.2 ± 6.3 [69–90]	78.5 ± 4.2 [71–87]	0.323
**Race**			
White	23	25	NS
African American	0	0	
Unknown	2	0	
**Ethnicity**			
Non-Hispanic	25	25	NS
Hispanic	0	0	
Unknown	0	0	
**Education**			
High school	3 (12%)	8 (32%)	0.089
College	8 (32%)	10 (40%)	
Post college	14 (56%)	7 (28%)	
**BMI, kg/m**^**2**^	22.3 ± 2.6 [18.7–26.8]	29.8 ± 4.5 [23.5–41.4]	< 0.001
**Waist circumference, cm**	75.7 ± 6.2 [66–89]	98.1 ± 10.1 [84–120]	< 0.001
**VAT area, cm**^**2**^	45.6 ± 12.5 [22.9–62.7]	177.5 ± 31.3 [137–246]	< 0.001
**Total energy intake, kcal/day**	1517 ± 577 [249–2762]	1349 ± 500 [673–2584]	0.429
**Healthy Eating Index (HEI) score**	77.2 ± 6.9 [66.1–95.3]	72.2 ± 10.3 [46.0–85.2]	0.051

^*^t-tests for continuous variables. Chi-square tests for categorical variables

**Fig. 2:**
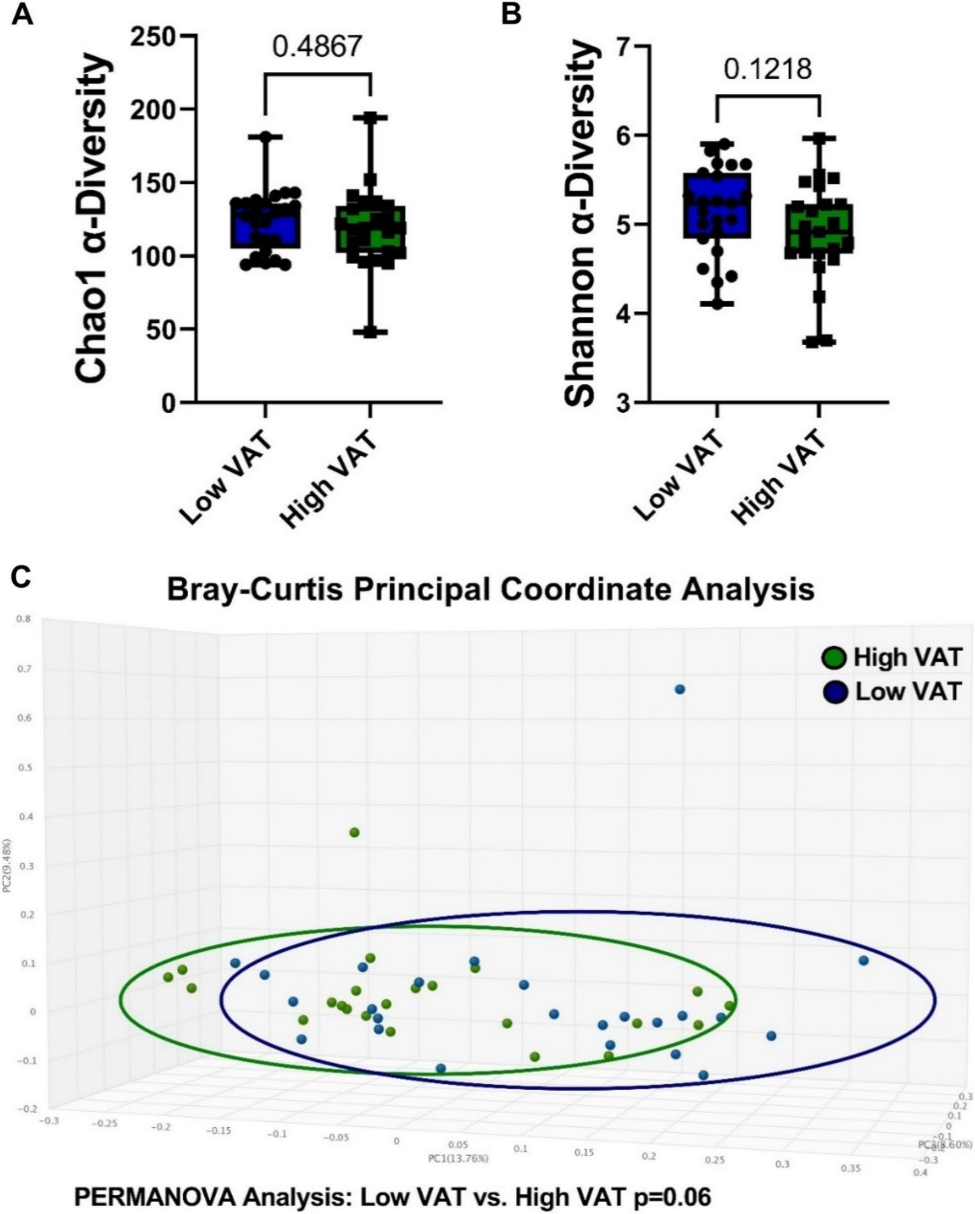
Visceral adipose tissue (VAT) area and gut microbiome diversity in elderly postmenopausal women. **A** There was no discernable difference in the Chao1 *α*-diversity measurements in women with differing VAT areas (*p* = 0.49). **B** Shannon *α*-diversity was not modified by visceral adiposity (*p* = 0.12). *n* = 23–24; two-tailed Mann–Whitney *U*-test. **C** Principal coordinate analysis (PCoA) Bray–Curtis *β*-diversity PERMANOVA analysis indicates a trend (*p* = 0.06) in dissimilarity between the gut bacterial populations in elderly postmenopausal women with high vs low VAT area. *n* = 23–24; PERMANOVA

There were no significant differences in the Chao1 (Fig. 2A) or Shannon (Fig. 2B) alpha-diversity indices between the VAT groups (*p* = 0.49 and *p* = 0.12, respectively). In the Bray–Curtis principal coordinate analysis, there was a trending difference in the gut bacterial communities between the high and low VAT groups (*p* = 0.06) based on the *β*-diversity dissimilarity index (Fig. 2C).

**Fig. 3:**
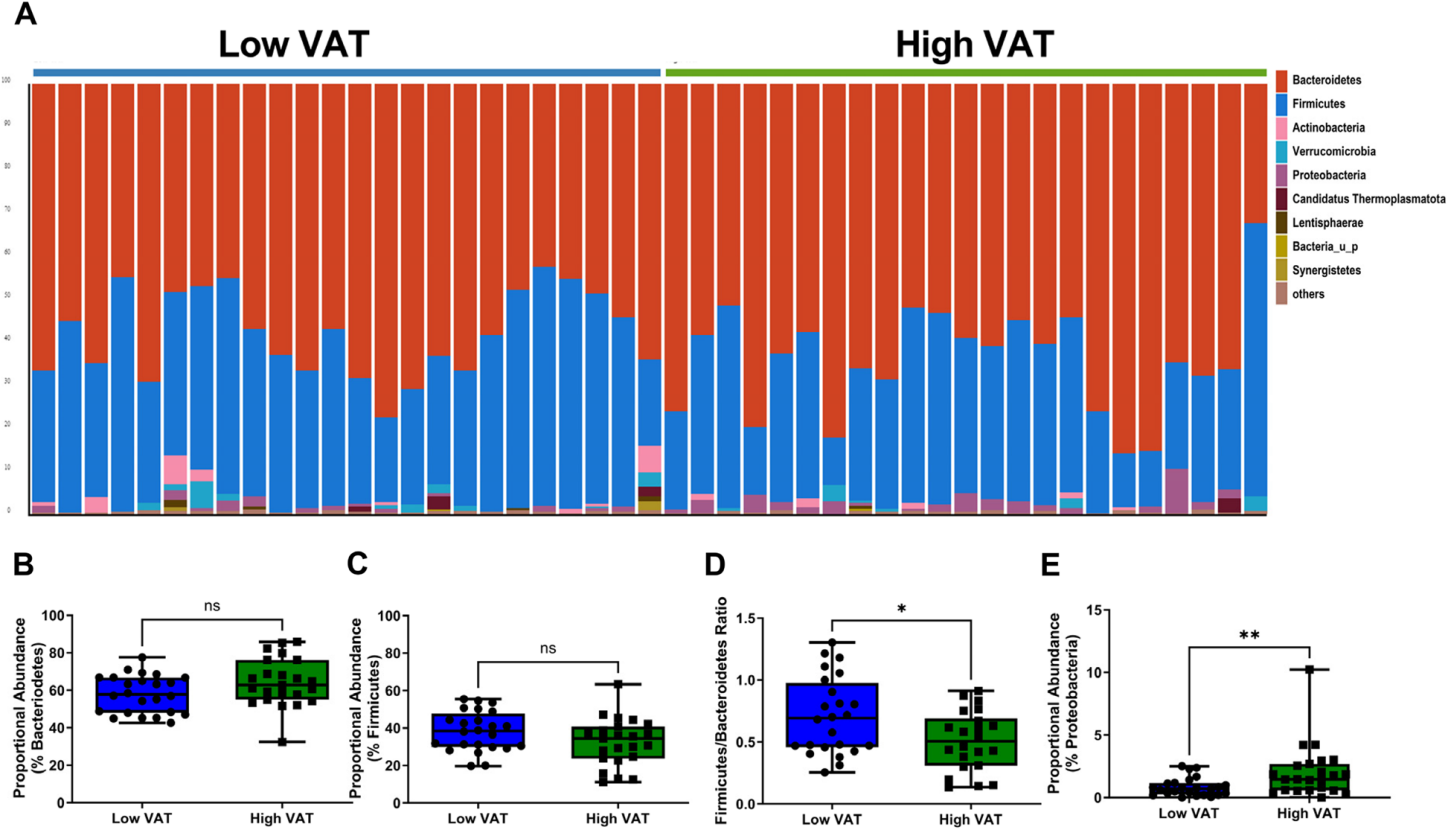
Adiposity is associated with key differences in phyla gut microbiome populations. Phyla level changes in gut bacterial microbiome populations in postmenopausal women with high or low VAT area. **A** Relative abundance of bacterial phyla in fecal samples is visualized by bar plots. Each bar represents the subjects aggregated by cohort and each colored box a bacterial taxon. The height of a color box represents the relative abundance of that organism within the sample. “Other” represents lower abundance taxa. **B** VAT area did not affect Bacteroidetes phylum proportional abundance. **C** There were no significant differences in the proportional abundance of Firmicutes in fecal samples from high VAT or low VAT postmenopausal women. **D** Postmenopausal women with low VAT area display an elevated Firmicutes/Bacteroidetes ratio when compared with participants with higher visceral adiposity. **E** Postmenopausal women with high VAT area display elevated proportional abundance of fecal Proteobacteria. *n* = 23–24. *p < 0.05, **p < 0.01 two-tailed Mann–Whitney *U*-test

The individual participant phyla proportional abundance can be observed in Fig. 3A. Each bar is an individual participant, each color represents a bacterial phylum, and the height of the bar represents the proportional abundance. While we observed no significant differences between low VAT and high VAT women regarding Bacteroidetes phylum (Fig. 3B) or Firmicutes phylum (Fig. 3C) proportional abundance, the Firmicutes/Bacteroidetes (F/B) ratio was significantly shifted by adiposity in postmenopausal women (*p* < 0.05; Fig. 3D). The F/B ratio was 0.71 ± 0.31 in postmenopausal women with low VAT area compared with a F/B ratio of 0.51 ± 0.24 in postmenopausal women with high VAT. We observed a significant enrichment in Proteobacteria phyla proportional abundance in postmenopausal women with high VAT (*p* < 0.01; Fig. 3E).

**Fig. 4:**
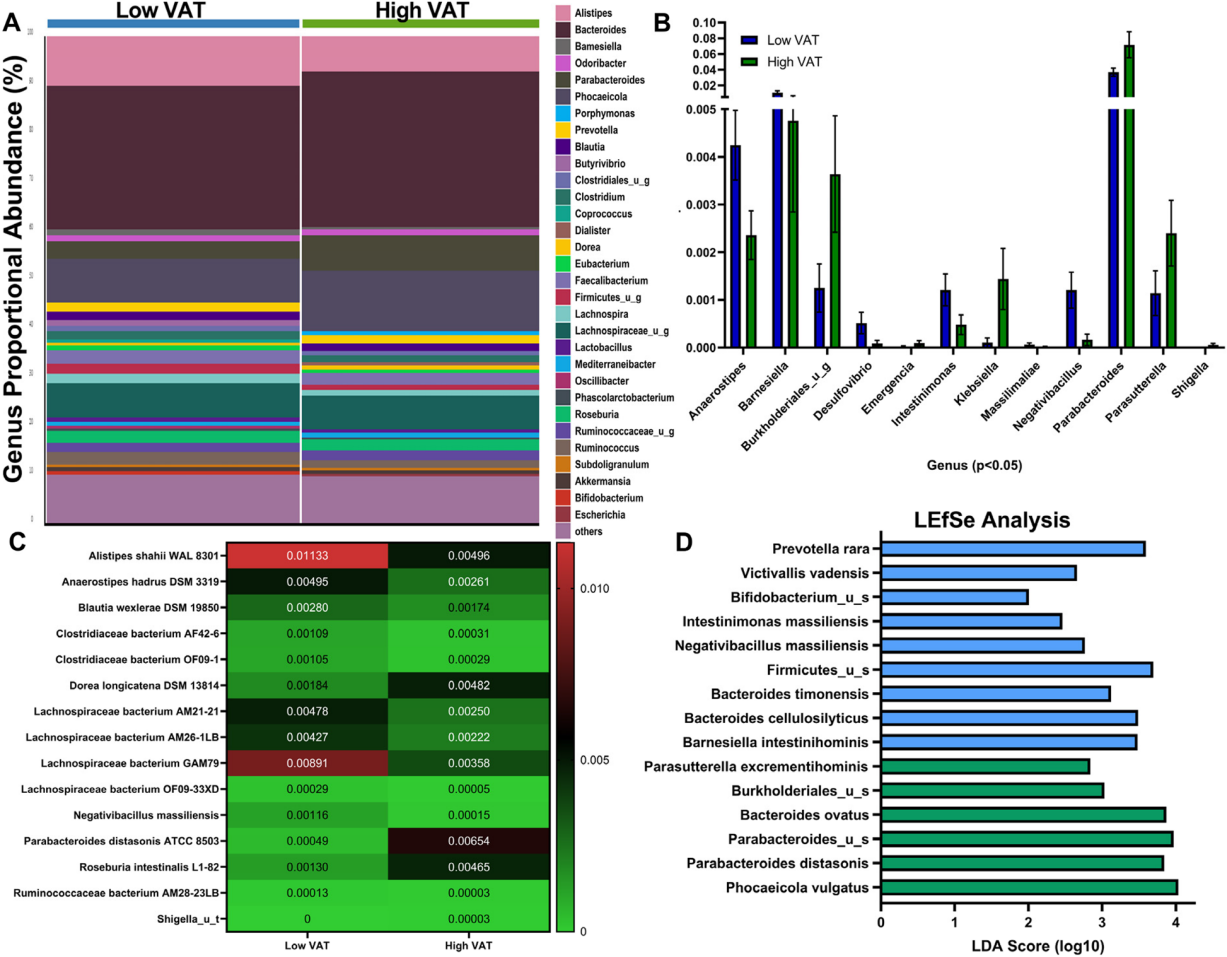
Postmenopausal women with differing VAT status display differential enrichment of bacterial genera, species, and strains within their gut microbiomes. **A** Relative abundance of bacterial genera in fecal samples is visualized by bar plots. Each bar represents the subjects aggregated by cohort and each colored box a bacterial taxon. The height of a color box represents the relative abundance of that organism within the group. “Other” represents lower abundance taxa. **B** Proportional abundance of significant genera by VAT area. **C** Heatmap of bacterial strains identified as significantly different by VAT area. Relative mean abundance is displayed in cell. *n* = 23–24. *p < 0.05, two-tailed Mann–Whitney *U*-test. **D** Linear discriminant analysis Effect Size (LEfSe) method identifying differentially enriched taxa by VAT area

Participant’s proportional abundance of gut bacteria genera were aggregated by VAT groupings and shown as a bar graph in Fig. 4A. Each bar is the aggregated participants from each group, each color represents a bacterial genus, and the height of the bar represents the relative proportional abundance of that organism within the group. “Other” represents lower abundance taxa. Of the 182 detectable genera of bacteria, 12 were significantly regulated by adiposity, with low VAT women expressing elevated proportional abundance of *Anaerostipes*, *Barnesiella*, *Desulfovibrio*, *Intestinimonas*, and *Negativibacillus* (Fig. 4B). Women with high VAT displayed enrichment of Burkholderiales of an unidentified species, *Emergencia*, *Klebsiella*, *Parabacteroides*, *Parasutterella*, and *Shigella* (Fig. 4B). At the strain levels, we identified the enrichment of three strains in the high VAT group (Fig. 4C): *Dorea longicatena* DSM 13814 (0.2% low VAT vs 0.5% high VAT), *Parabacteroides distasonis* ATCC 8503 (0.05% low VAT vs 0.7% high VAT), and *Roseburia intestinalis* L1-82 (0.1% low VAT vs 0.5% high VAT). We identified 11 strains enriched in the low VAT group (Fig. 4C): *Alistipes shahii* WAL8301 (1.1% low VAT vs 0.5% high VAT), *Anaerostipes hadrus* DSM 3319 (0.5% low VAT vs 0.3% high VAT), *Blautia wexlerae* DSM 19850 (0.3% low VAT vs 0.2% high VAT), *Negativibacillus massiliensis* (1.1% low VAT vs 0.02% high VAT), Clostridiaceae bacterium AF42-6 (0.1% low VAT vs 0.03% high VAT), Clostridiaceae bacterium OF09-1 (0.1% low VAT vs 0.03% high VAT), Lachnospiraceae bacterium AM21-21 (0.5% low VAT vs 0.3% high VAT), Lachnospiraceae bacterium AM26-1LB (0.5% low VAT vs. 0.2% high VAT), Lachnospiraceae bacterium GAM79 (0.9% low VAT vs. 0.4% high VAT), and Lachnospiraceae bacterium OF09-33XD (0.03% low VAT vs. 0.01% high VAT). Linear discriminant analysis effect size (LEfSe; Fig. 4D) at the identified species level determined *Phocaeicola vulgatus* (*LDA* = 4.04; *p* = 0.04) in high VAT group and *Prevotella rara* (*LDA* = 3.59; *p* = 0.04) in low VAT group as the most differentially enriched taxa by VAT groups.

**Fig. 5:**
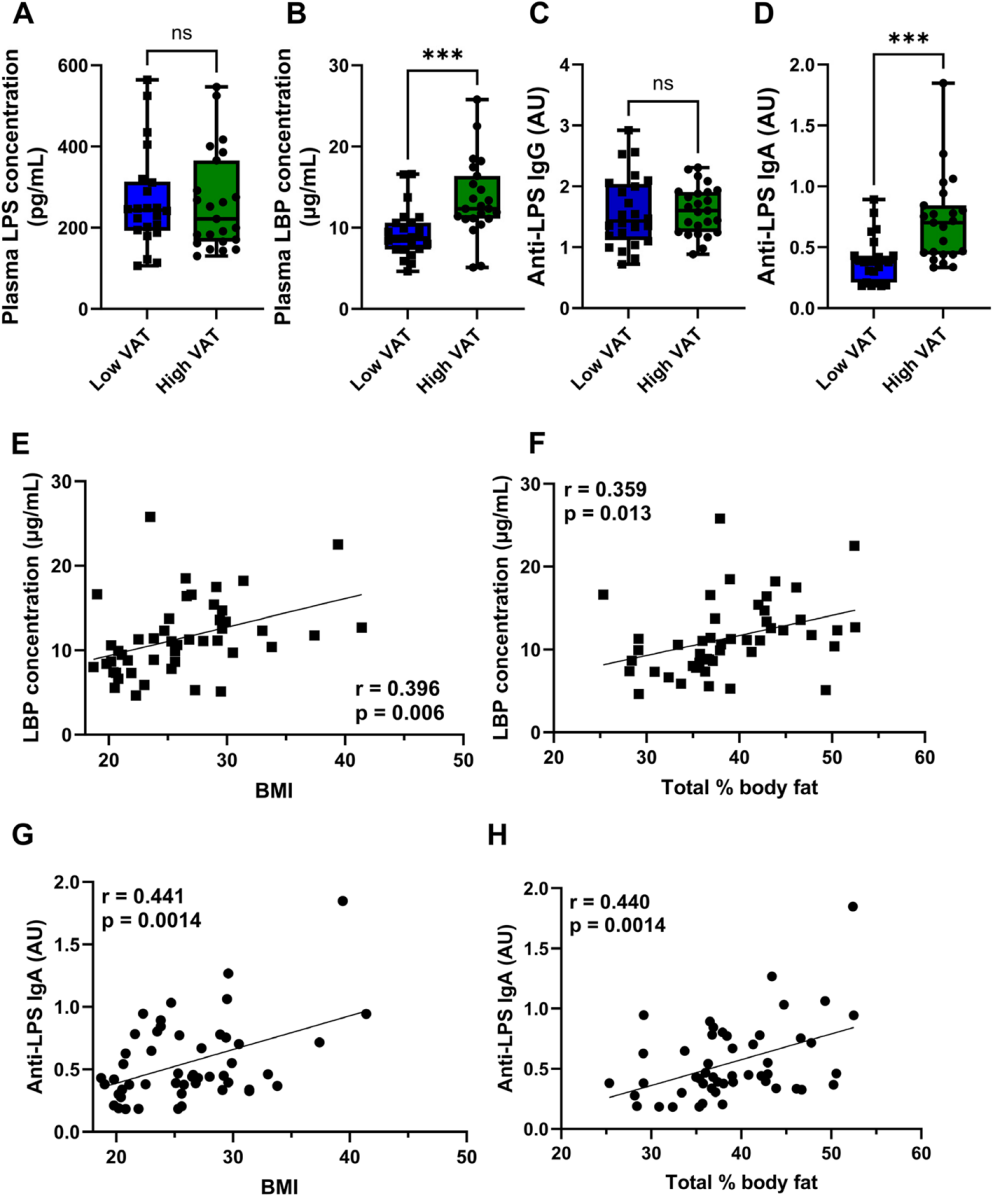
Circulating LPS-binding protein and anti-LPS IgA antibody concentrations correlates with body fat mass and BMI indexes. **A** Plasma LPS (pg/mL) was not significantly modulated by VAT area. **B** Plasma LPS-binding protein (LBP) was elevated in the high VAT group compared with the low VAT group (13.6 µg/mL vs 9.3 µg/mL). **C** Plasma anti-LPS IgG was not shifted by VAT area. **D** Plasma anti-LPS IgA was elevated in high VAT group when compared with low VAT group (0.69 AU vs. 0.39 AU). *n* = 23–24, *p < 0.05 Welch’s *t*-test. **E** Plasma LBP concentrations significantly correlated with participant BMI (*r* = 0.396; *p* = 0.006). **F** Plasma LBP concentrations significantly correlated with participant % body fat (*r* = 0.359; *p* = 0.013). **E** Plasma anti-LPS IgA concentrations significantly correlated with participant BMI (*r* = 0.441; *p* = 0.0014). **F** Plasma anti-LPS IgA concentrations significantly correlated with participant % body fat (*r* = 0.440; *p* = 0.0014). *n* = 23–24. Pearson’s correlation coefficient (r)

We did not observe any differences between VAT groups according to plasma LPS concentrations (Fig. 5A); however, plasma LBP (Fig. 5B) and anti-LPS IgA antibodies (Fig. 5D) were found in higher concentration in postmenopausal women with high VAT compared to low VAT. There were no significant differences in plasma anti-LPS IgG antibody concentrations (Fig. 5C). LBP was positively and significantly correlated with BMI and % body fat (*r* = 0.359 and 0.396, respectively, both *p* < 0.013) (Fig. 5E, F). Similarly, anti-LPS IgA was positively correlated with BMI and % body fat (*r* = 0.441 and 0.440, respectively, both *p* < 0.0014) (Fig. 5G, H). Moreover, anti-LTA IgA and anti-flagellin IgA antibodies were significantly higher in the high VAT group compared to the low VAT group (Supplemental Figure S4 B and D). The anti-LTA IgG and anti-flagellin IgG antibodies were not different between the two VAT groups (Supplemental Figure S4 A and C).

**Fig. 6:**
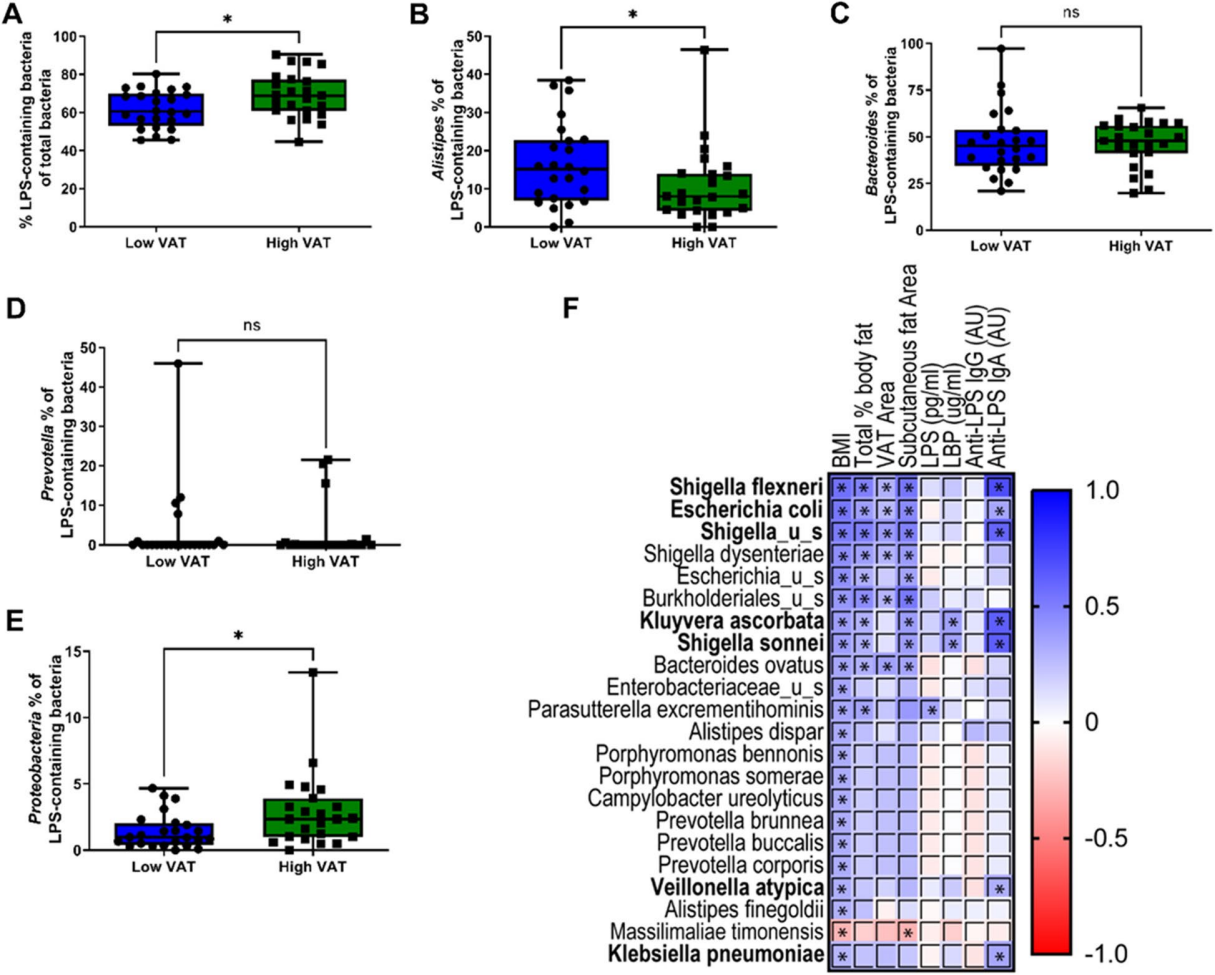
LPS-expressing bacterial species correlate with both body adiposity measurements and inflammatory indicators in elderly postmenopausal women. **A** LPS-expressing bacteria proportional abundance by lpxA and lpxB gene expression in low VAT and high VAT women. **B** % *Alistipes* of LPS-expressing microbes is significantly higher in low VAT women than in high VAT women. **C** No difference in proportional abundance % Bacteroides of LPS-expressing microbes in fecal samples between groups. **D** No difference in proportional abundance % *Prevotella* of LPS-expressing microbes in fecal samples between groups. **E** Elevated proportional abundance of Proteobacteria of the LPS-expressing microbes is observed in high VAT area participants when compared with low VAT women. **F** Correlation matrix of LPS-containing gut bacteria that are significantly associated with body anthropometric and gut inflammation markers. Bacteria are presented in ascending order of *p*-value for association with BMI. Bacteria in bold are those that significantly correlated with both BMI and anti-LPS IgA production

All 431 species/taxonomic units of bacteria identified in the participants were mined for LPS expression as defined by lpxA and lpxB gene expression. The percentage of LPS-containing bacteria was significantly higher in the high VAT group (69.1%) compared to the low VAT group (61.8%) (Fig. 6A). Accumulating evidence suggests the presence of inhibitory forms of LPS that act as antagonists on the toll-like receptor-4 (TLR4). Members of the order Bacteroidales (*Alistipes*, *Bacteroides*, and *Prevotella*) are known to possess this inhibitory form of LPS, while the phylum Proteobacteria possesses the prototypical stimulatory LPS [36, 37]. The *Alistipes* proportion of LPS-containing bacteria was significantly lower in the high VAT group (Fig. 6B). Bacteroides (Fig. 6C) and *Prevotella* (Fig. 6D), however, did not show significant differences. The Proteobacteria proportion of LPS-containing bacteria was significantly higher in the high VAT group (Fig. 6E).

Next, we determined which LPS-containing bacterial species are associated with adiposity and gut inflammatory responses. Twenty-two bacteria showed significant associations with BMI (*p* < 0.05) which were depicted in a correlation matrix in ascending order of their *p*-values (Fig. 6F). Of those bacteria, seven species (*Shigella flexneri*, *Escherichia coli*, *Shigella_u_s*, *Kluyvera ascorbata*, *Shigella sonnei*, *Veillonella atypica*, and *Klebsiella pneumoniae*) associated with plasma anti-LPS IgA levels (Fig. 6F; bacteria shown in bold), and none associated with anti-LPS IgG. Furthermore, *K. ascorbata* and *S. sonnei* are also significantly associated with BMI, % body fat, subcutaneous body fat, plasma anti-LPS IgA, and plasma LBP concentrations.

Of the LTA-expressing bacteria, we only found eight bacteria that were significantly associated with BMI (*p* < 0.05) which were depicted in a correlation matrix in ascending order of their *p*-values (Supplemental Figure S5A). However, none of these bacteria seemed to be immunostimulatory, hence the absence of associations with either anti-LTA IgG or anti-LTA IgA (Supplemental Figure S5A). On the other hand, three of the nine motile flagellated bacteria that were significantly associated with BMI (*p* < 0.05) stimulated the production of anti-flagellin IgA (but not IgG) as shown in the correlation matrix (Supplemental Figure S5B).

**Fig. 7:**
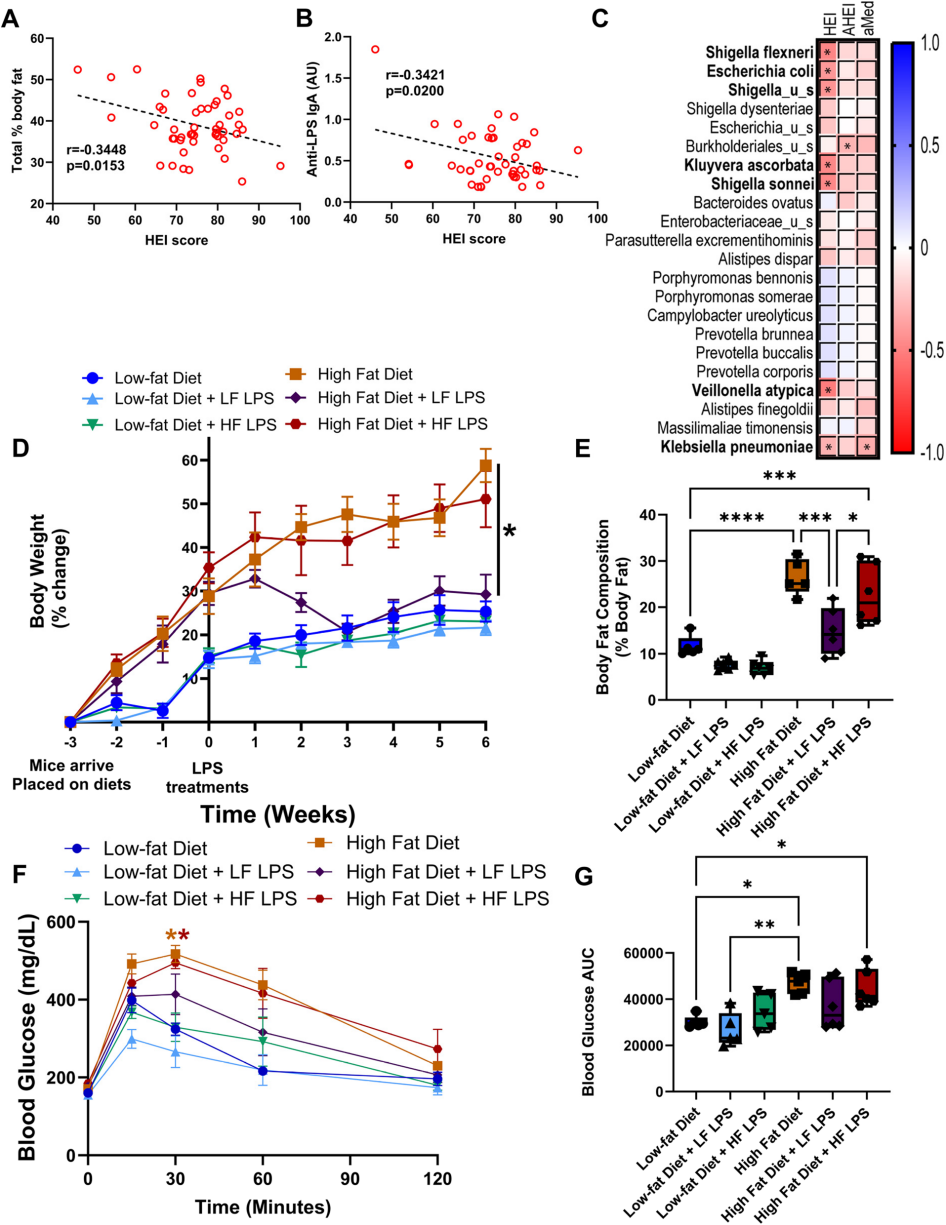
Diet-associated LPS differentially modulates metabolic outcomes. **A** Healthy Eating Index 2015 (HEI-2015) scores negatively correlated with total % body fat (Pearson’s correlation coefficient *r* =  − 0.3448, *p* = 0.0153, *n* = 50). **B** HEI-2015 score negatively correlated with plasma anti-LPS IgA antibody concentrations (Pearson’s correlation coefficient *r* =  − 0.3421, *p* = 0.0200, *n* = 50). **C** Correlation matrix of LPS-containing gut bacteria that are significantly associated with body anthropometric and HEI-2015. Bacteria in bold are those that significantly correlated with both BMI and anti-LPS IgA production. *p < 0.05, *n* = 50. **D**. Female C57BL/6 mouse body weight of low-fat and high-fat diet-fed mice given saline and LPS interventions modeling metabolic endotoxemia concentrations. LPS was isolated from fecal samples collected from low-fat diet-fed mice (LF-LPS) and high-fat diet-fed mice (HF-LPS); *n* = 5–6, *p < 0.05. **E** Body fat composition of low-fat diet-fed and high-fat diet-fed saline and diet-fecal-derived LPS-treated animals at the end of the study. **F** Blood glucose concentrations in low-fat diet-fed and high-fat diet-fed saline and diet-fecal-derived LPS-treated animals at the end of the study during a glucose tolerance test. *p < 0.05 compared to low-fat diet groups, *colored to group. **G** Blood glucose area under the curve (AUC) of low-fat diet-fed and high-fat diet-fed saline and diet-fecal-derived LPS-treated animals at the end of the study. *n* = 5–6; *p < 0.05

Diet can shape the composition of the gut microbiome [24], and high-energy intake is a risk factor for obesity. HEI-2015 scores, indicating diet quality, were available for each of the participants within the study. We show that HEI-2015 scores negatively correlated with participant % body fat composition (Fig. 7A) and plasma anti-LPS IgA concentration (Fig. 7B). Moreover, the seven bacterial species (*S. flexneri*, *E. coli*, *Shigella_u_s*, *Kluyvera ascorbate*, *S. sonnei*, *V. atypica*, and *K. pneumoniae*) positively associated with adiposity and plasma anti-LPS IgA levels are negatively correlated with the HEI-2015 score (Fig. 7C) indicating that poor dietary patterns are associated with immunogenic forms of LPS and metabolic endotoxemia.

To mechanistically investigate the impact of diet on LPS-mediated metabolic endotoxemia, we treated low-fat and high-fat diet-fed female C57BL/6 mice with saline, low-fat diet-derived fecal isolated LPS, or high-fat diet-derived fecal-isolated LPS for 6 weeks. High-fat diet consumption in mice elevates fecal Proteobacteria (highly acylated LPS), while low-fat diets favor under-acylated LPS-expressing Bacteroidetes bacterial populations [38, 39]. While LPS treatments did not impact low-fat diet-fed animals, low-fat diet-derived fecal isolated LPS administration in high-fat diet-fed animals reduced body weight (Fig. 7D), decreased total % body fat (Fig. 7E), and improved glucose tolerance test outcomes (Fig. 7F and G), suggesting acylation status of LPS isoforms may impact the development of adverse metabolic outcomes observed in obesity.

## Discussion

Our study links the gut microbiome composition with both abdominal adiposity (measured by DXA scans) and circulating gut permeability inflammation-related biomarkers in postmenopausal women. Further, our study identified a higher prevalence of LPS-containing bacteria in postmenopausal women with higher VAT area and associated specific species that may contribute to LPS immunostimulation. Scores on the HEI-2015 were negatively associated with percent body fat and plasma anti-LPS IgA concentrations, supporting the hypothesis that poor diet quality leads to visceral adiposity perhaps through its influence on the composition of the gut microbiome. Additionally, our animal work suggests that LPS isoforms differentially influence the development of adverse metabolic outcomes observed in obesity.

Aging is associated with changes in the gut microbiome F/B ratio. The F/B ratio drastically increases during infancy, peaks in adulthood, and then decreases in the elderly [40–43]. This decrease in the F/B ratio is very drastic and well-documented for older age groups (i.e., > 70 years of age) which is similar to the age of our cohort [40–43]. Studies that considered lower age cutoffs (i.e., 50–70 years of age) showed higher F/B ratios and/or insignificant differences [44–46]. This is true for mice, as well, where aged mice (≥ 20 months) showed a marked decline in F/B ratios [44, 47–49], whereas results from middle-aged mice (10–20 months) were less evident and/or contradictory [50–52].

Obesity, on the other hand, is a factor that has been reported to increase the F/B ratio [4, 53], suggesting that obesity and aging display counter-regulatory effects on F/B ratio. Worthy of mention is the lack of reproducibility in human cohorts where some have reported no changes or even a decrease in the F/B ratio in obese individuals [15]. Herein, we show that abdominal adiposity is associated with a decreased F/B ratio in elderly postmenopausal women. These data suggest that obesity, even among a subset of elderly women, is associated inversely with the F/B ratio, thus potentially inducing dysbiosis independent of aging. This is supported by two studies on frail elderly subjects in hospitals and long-term residential care facilities (mean ages > 70 years) which had lower F/B ratios than similar aged healthier community-dwelling subjects [54, 55]. Another study from urbanized towns in Korea showed lower F/B ratios in elderly compared to healthier counterparts and centenarians from longevity villages [55]. The high VAT group in our study showed an enrichment of Proteobacteria which is an indicator of dysbiosis and unhealthy aging documented in hospitalized elderly [55]. These gram-negative bacteria contain immunostimulatory LPS capable of inducing strong inflammatory responses [37, 56]. This is corroborated by multiple lines of evidence showing an expansion of Proteobacteria and its implication in inflammatory conditions such as inflammatory bowel disease (IBD) and metabolic disorders [57, 58].

Obesity is known to chronically and modestly elevate LPS/endotoxin levels causing a condition termed “metabolic endotoxemia” [59–61]. LPS is the major structural glycolipid component of the outer membranes in gram-negative bacteria. Obese subjects that underwent bariatric surgery showed a reduction in their plasma LPS levels 1-year post-operation [62], suggesting that weight loss improved gut barrier integrity. Elevated plasma LPS is associated with poor intestinal health markers such as reduced villi length, decreased goblet cell number, and increased muscularis thickness in a nonhuman primate model [63], indicating that plasma LPS is an effective biomarker for gut health. Yet, we did not observe any differences between high and low VAT groups in plasma LPS concentrations. This could be due to the fact that aging-induced changes in the gut microbiome increase gut leakiness due to reduced butyrate production and compromised mucin barriers [52]. This increased gut leakiness in our sample of postmenopausal women may have increased LPS levels to a limit beyond which adiposity cannot significantly produce any noticeable differences. This is corroborated by the observation that in our study, LPS concentrations in both lean and obese groups are triple the values reported in a study of middle-aged participants [62].

LPS comes in many different isoforms regarding its structural features that collectively determine immunogenic potential. LPS structure is composed of three major moieties: lipid A, core oligosaccharide, and O-antigen [56]. The O-antigen is the most surface-exposed part which plays a critical role in immune evasion by preventing adhesion/phagocytosis and protecting against bactericidal responses. Lipid A is the most-inner part that is responsible for the immunogenicity of LPS. Lipid A is an acylated and phosphorylated disaccharide of glucosamine that has varying length, number, distribution, and saturation of its fatty acid side chains [34, 56, 64, 65]. The general notion is that the immunogenicity of lipid A increases as the number of phosphate groups and acyl chains increases [34, 36, 56, 66]. Emerging studies even argue that endotoxemia could be metabolically beneficial based on the lipid A structure [34, 37]. For instance, under-acylated LPS counteracted the dysglycemia and inflammation caused by an equal dose of endotoxin units of the prototypic hexa-acylated *E. coli* LPS [34].

In our study, the principal coordinate analysis suggests a dissimilar gut composition based on VAT. Moreover, women with differing VAT status displayed differential enrichment of bacterial genera, species, and strains within their gut microbiomes. It might be the case that the two VAT groups, although having similar plasma LPS levels, differ in their LPS immunogenicity and inflammatory responses. Indeed, plasma LBP and anti-LPS IgA antibodies were significantly elevated in plasma samples from the high VAT group in comparison to the low VAT group. These two markers also significantly correlated with other measures of adiposity such as BMI and total % body fat. LBP binding to LPS contributes to the effective recognition and enhanced sensitivity of LPS to its receptor: toll-like receptor-4 (TLR4). LBP is a lipid transferase that catalyzes the extraction and the subsequent presentation of lipid A from bacterial membranes to TLR4 [67]. Blockade of LBP attenuated the inflammatory effects of LPS in animal studies [68, 69]. Plasma levels of LBP have also been reported to correlate with adiposity measures and insulin resistance [62, 70]. IgA antibody, on the other hand, is the most abundantly produced isotype by plasma cells on mucosal surfaces, and it encompasses 15% of antibodies in the serum [71]. IgA in the gut is primarily induced by microbiota and food antigens [72]. Studies have shown a direct relationship between the pathogenicity of an organism and its relative coating levels with IgA [73, 74]. For instance, high levels of IgA coating identified inflammation-inducing bacteria (colitogenic bacteria) in the colons of IBD patients [74]. Since IgG is mainly secreted by plasma cells in the blood [71], the absence of significant increase in anti-LPS IgG levels in the high VAT group suggests that the bloodstream is not the compartment in which plasma cells have encountered and reacted to LPS. On the other hand, the significant increase in anti-LPS IgA levels in the high VAT group may be reflective of mucosal immunity and GI inflammation [75, 76]. Taken together, the higher levels of LBP and anti-LPS IgA in the high VAT group indicate the presence of immunogenic LPS in the gut that may induce metabolically detrimental endotoxemia.

The high VAT group harbored more LPS-containing bacteria than the low VAT group. More importantly, the proportion of Proteobacteria (phyla) within the LPS-containing bacteria was higher in the high VAT group, while the proportion of *Alistipes* (genus) was lower. In other words, the ratio of bacteria with immunogenic LPS (Proteobacteria) to bacteria with immunosuppressive LPS (Alistipes) is higher in the high VAT group in comparison to the low VAT group. The immunostimulatory and inhibitory effects of LPS from Proteobacteria and *Alistipes*, respectively, are well-described and consistent with their acylation patterns, where members of Proteobacteria are hexa-acylated and members of Bacteroidales (*Alistipes*, *Bacteroides*, and *Prevotella*) are penta- and tetra-acylated [37]. Twenty-two LPS-containing bacteria in our study showed significant positive associations with BMI which are depicted in the correlation matrix. Out of them, seven bacteria showed a significant positive association with anti-LPS IgA as well. Not surprisingly, all of those shared correlates (between BMI and anti-LPS IgA) are Proteobacteria with the exception of *V. atypica* which is an atypical gram-negative Firmicute that possesses a typical proteobacterial LPS biosynthesis system [77]. In summary, all adiposity-associated bacteria that elicited an immune response (anti-LPS IgA) conferred an immunogenic form of LPS. This strongly indicates a causal link between these immunogenic bacteria with prototypical lipid A structure and obesity-associated gut inflammation in older women.

In vivo experiments indicated that low-fat diet-derived fecal LPS administered to high-fat diet-fed animals caused a reduction in body weight, a decrease in total % body fat, and an improvement of glucose tolerance test outcomes. This improvement in metabolic outcomes by low-fat diet-derived fecal LPS could be attributed to the predominance of under-acylated forms of LPS in the guts of lean mice which are known to compete with immunostimulatory forms of LPS enriched in obese mice [34, 37]. Indeed, feces from healthy individuals were shown to be enriched in under-acylated forms of LPS and, intriguingly, silence the pro-inflammatory TLR4 signaling [37]. However, high-fat diet-derived fecal LPS did not result in significant perturbations in metabolic outcomes. This contrasts with a study showing *E. coli* LPS (hexa-acylated) to cause dysglycemia and inflammation in both obese and lean mice [34]. Differences with their study design including the use of male mice and purified LPS from a single bacterium may explain some of the divergent results, suggesting further investigation into sex differences of endotoxemia and its metabolic outcomes are needed.

LTA is the major structural constituent of the cell walls of gram-positive bacteria that is recognized by TLR2, while flagellin is the protein subunit of the locomotory organ of flagellates that is recognized by TLR5 [78]. Although we have seen an association between LTA IgA levels and adiposity, none of the LTA-expressing bacteria was significantly correlated with anti-LTA antibodies. On the other hand, anti-flagellin IgA antibodies were significantly elevated in the high VAT group in comparison to the low VAT group. Nine motile flagellin-expressing bacteria showed a significant association with adiposity. Three of those nine motile flagellates (*Campylobacter ureolyticus*, Clostridiales Family XIII. Incertae Sedis_u_s, and *Fenollaria timonensis*) also showed a significant association with plasma anti-flagellin IgA levels. The correlation with *Campylobacter ureolyticus* abundance is not surprising since it is a known inflammation-inducing pathogen associated with the development of gastroenteritis [79, 80]. However, Clostridiales Family XIII. Incertae Sedis_u_s and *Fenollaria timonensis* are not well-studied, and it is unknown at this point if their association with anti-flagellin IgA antibodies could be causal. Yet, importantly, our study shows that obesity-associated gut inflammation in postmenopausal women is not merely LPS-mediated but potentially a flagellin-mediated response as well.

It is critical to mention that we specifically selected a sample of women that were not on medications in order to reduce potential confounders. Previous literature in preclinical and clinical models indicate diabetes medications (metformin), hypertension drugs (ACEi and ARBs), and lipid-lowering therapeutics (statins) can modify the gut microbiome [81–83]. Therefore, our study represents an age-matched, sex-matched, and comorbidity-controlled investigation on adiposity influencing the gut microbiome. However, this also indicates that our high VAT group also represents a potentially metabolically healthy obese group. Metabolic syndrome is defined as ≥ 3 clinical indicators of metabolic dysfunction (fasting glucose ≥ 100 mg/dL, waist circumference > 88 cm, triglycerides > 150 mg/dL, *HDL* < 40 mg/dL, *BP* ≥ 130/85 [84]). Future studies will investigate the impact of metabolic dysfunction in metabolically healthy and metabolically unhealthy obese postmenopausal women.

## Conclusion

Our study investigated the impact of DXA-derived visceral adiposity, instead of BMI, as a measure of obesity and the associations with the gut bacterial microbiome population and gut inflammation markers. We identified that adiposity is negatively associated with diet quality and the F/B ratio in elderly postmenopausal women with enrichment in pro-inflammatory Proteobacteria. We show that low visceral adiposity is associated with a more commensal microbiome with elevated SCFA-producing microbe proportional abundances. We also identified that plasma LBP and anti-LPS IgA antibodies correlate positively with body fat composition and are positively associated with immunogenic Proteobacteria species, and anti-LPS IgA is negatively correlated with diet quality. Lastly, to provide mechanistic insight, we demonstrate that administration of LPS derived from feces of low-fat diet-fed mice improved metabolic parameters in high-fat diet-fed female C57BL/6 mice, suggesting LPS acylation status is an important factor for detrimental endotoxemia. Taken together, our data suggest that visceral adiposity in postmenopausal women is associated with poor diet quality and gut inflammation, potentially contributing to unhealthy aging.

## Supplementary Information

Supplementary Material 1: Supplemental data: ELISA data. Alpha diversity. Species.

Supplementary Material 2: Supplemental Figure S1. Group 2 (Red box/blue circles): Highest Year 17 VAT +VAT gain since Year 5 subjects (*n*=25). Group 1 (Black box/red circles): Lowest Year 17 VAT +VAT loss since Year 5 subjects (*n*=25). 4 underweight (BMI<18.5; grey #) in lower left corner. Supplemental Figure S2. 3 participants had detectable levels of either amoxicillin (antiobiotic targeting Gram-positive bacteria) or clindamycin (antibiotic targeting both Gram-positive and Gram-negative bacteria) and were excluded from further analysis. Supplemental Figure S3. Read statistics. Metagenomic read depth varied from 8.4M to 39M per sample. Supplemental Figure S4. Anti- Lipoteichoic acid (LTA) IgA and anti-flagellin IgA levels increased significantly in the high VAT group. A. No significant difference in anti-LTA IgG levels between the VAT groups. B. High VAT group showed significantly higher anti-LTA IgA. C. No significant difference in the levels of anti-flagellin IgG between the VAT groups. D. Anti-flagellin IgA was significantly higher in the high VAT group. Supplemental Figure S5. Select adiposity-associated motile bacteria correlated with anti-flagellin IgA while no LTA-containing bacteria correlated with anti-LTA antibodies. A. Correlation matrix of LTA-containing bacteria that are significantly associated with body anthropometric measures. Bacteria are presented in ascending order of *p* value for association with BMI (*p*<0.05). No bacteria showed any correlation with anti-LTA antibodies. B. Correlation matrix of motile bacteria that are significantly associated with body anthropometric measures (*p*<0.05). Bacteria in bold are those that significantly correlated with both BMI and anti-flagellin IgA production.

## Data Availability

All data are provided in the supplementary materials and the “ Material and methods” section. Excel spreadsheets for all the raw data are uploaded as supplemental data.
