# Supplementary material for: Visceral adiposity in postmenopausal women is associated with a pro-inflammatory gut microbiome and immunogenic metabolic endotoxemia
Source: Microbiome. 2024 Oct 4;12:192. doi: 10.1186/s40168-024-01901-1 (PMC11453046; doi:10.1186/s40168-024-01901-1)

**Supplemental Figure S1.**

Group 2 (Red box/blue circles): Highest Year 17 VAT +VAT gain since Year 5 subjects (n=25)

Group 1 (Black box/red circles): Lowest Year 17 VAT +VAT loss since Year 5 subjects (n=25)

4 underweight (BMI<18.5; grey #) in lower left corner

**Supplemental Figure S2.**


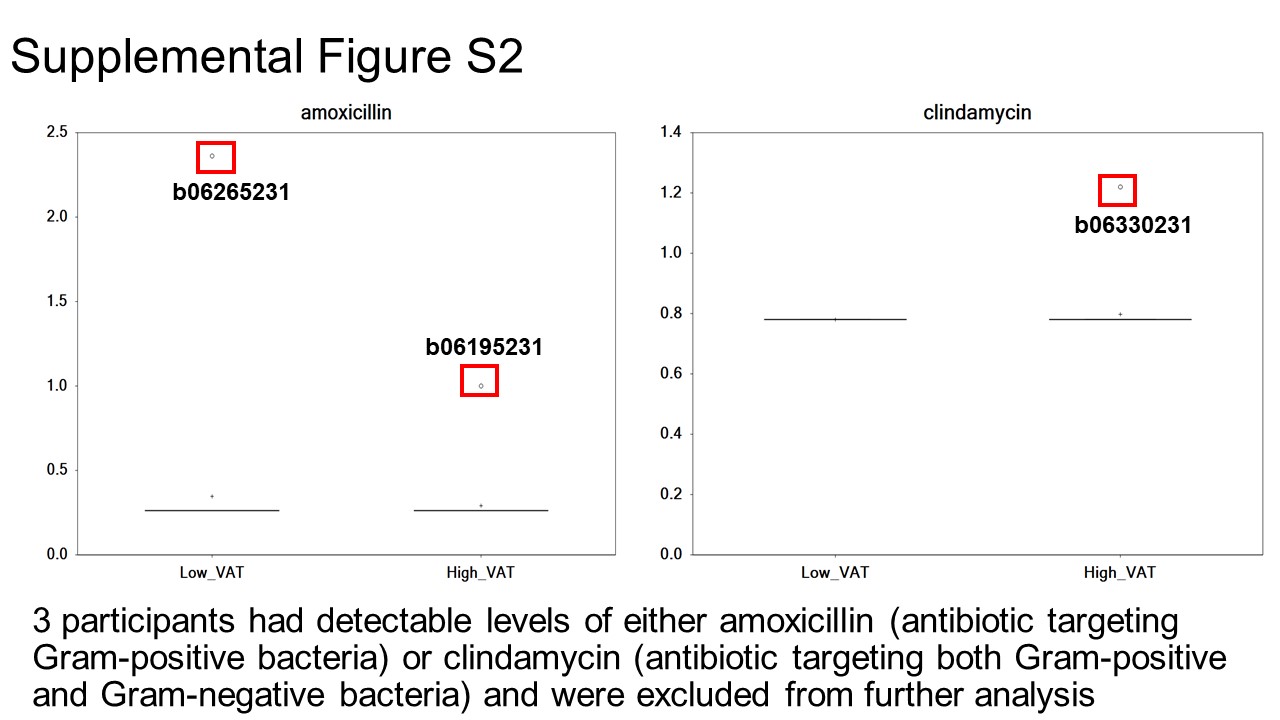


**Supplemental Figure S3.** Read statistics. Metagenomic read depth varied from 8.4M to 39M per sample.

**Supplemental Figure S4.** Anti- Lipoteichoic acid (LTA) IgA and anti-flagellin IgA levels increased significantly in the high VAT group. **A.** No significant difference in anti-LTA IgG levels between the VAT groups. **B.** High VAT group showed significantly higher anti-LTA IgA. **C.** No significant difference in the levels of anti-flagellin IgG between the VAT groups. **D.** Anti-flagellin IgA was significantly higher in the high VAT group.


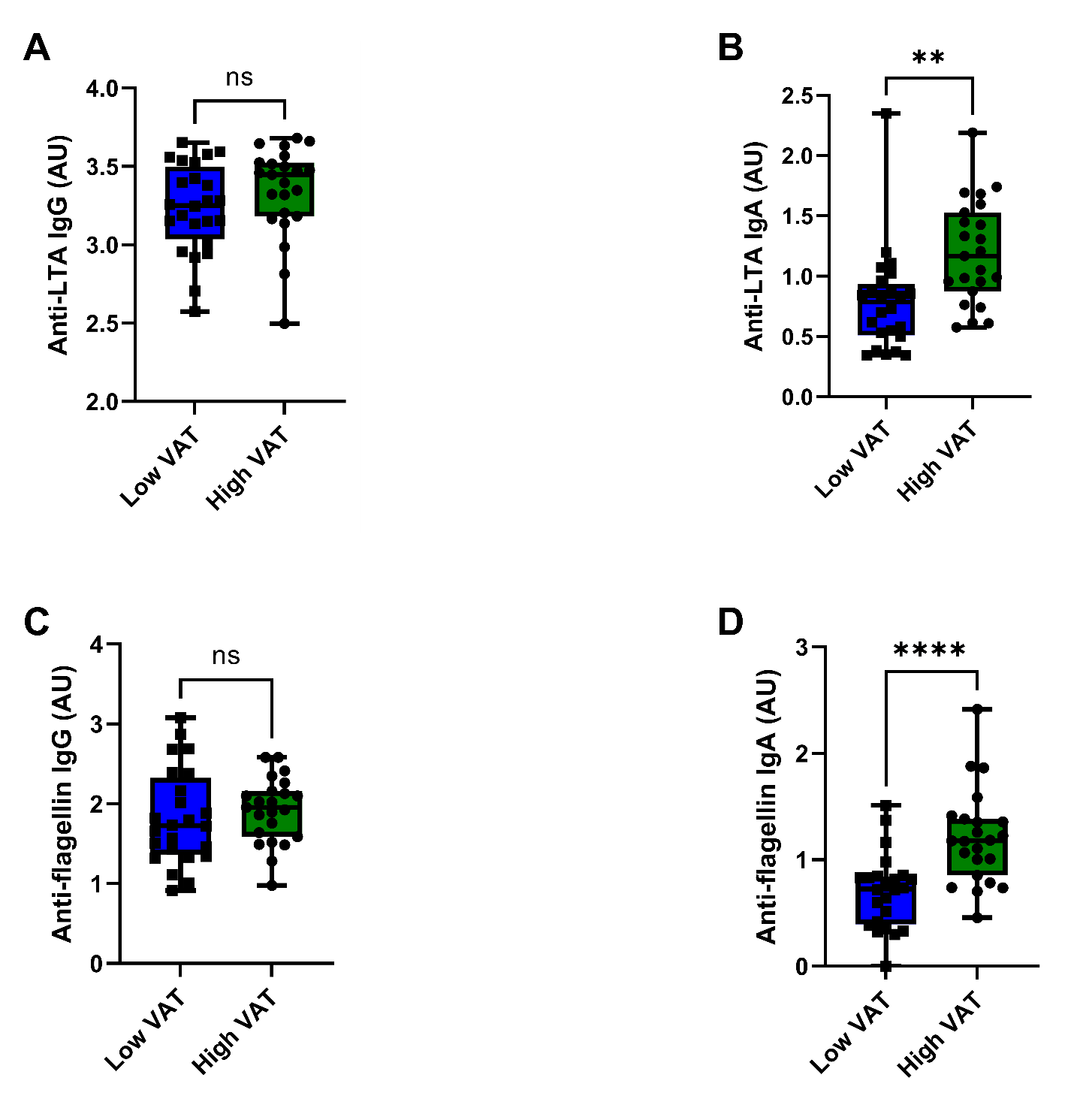


**Supplemental Figure S5.** Select adiposity-associated motile bacteria correlated with anti-flagellin IgA while no LTA-containing bacteria correlated with anti-LTA antibodies. **A.** Correlation matrix of LTA-containing bacteria that are significantly associated with body anthropometric measures. Bacteria are presented in ascending order of p value for association with BMI (p<0.05). No bacteria showed any correlation with anti-LTA antibodies. **B.** Correlation matrix of motile bacteria that are significantly associated with body anthropometric measures (p<0.05). Bacteria in bold are those that significantly correlated with both BMI and anti-flagellin IgA production.


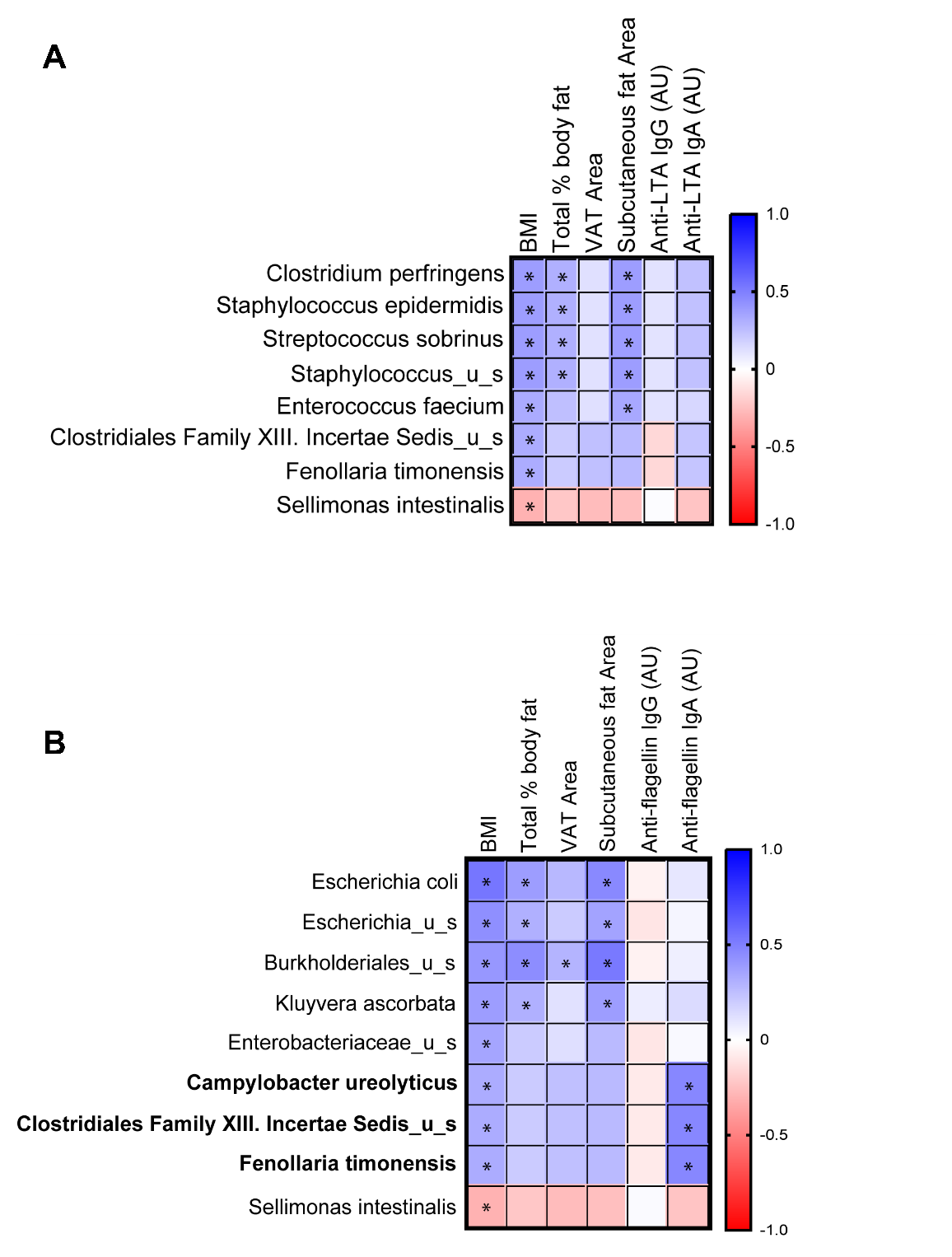

Supplement: Supplementary file 2 — Supplementary Material 1: Supplemental data: ELISA data. Alpha diversity. Species. [file 40168_2024_1901_MOESM1_ESM.docx]
